# A Novel Accuracy and Similarity Search Structure Based on Parallel Bloom Filters

**DOI:** 10.1155/2016/4075257

**Published:** 2016-12-07

**Authors:** Chunyan Shuai, Hengcheng Yang, Xin Ouyang, Siqi Li, Zheng Chen

**Affiliations:** ^1^Faculty of Electric Power Engineering, Kunming University of Science and Technology, Kunming 650051, China; ^2^Faculty of Information Engineering and Automation, Kunming University of Science and Technology, Kunming 650051, China; ^3^Faculty of Transportation Engineering, Kunming University of Science and Technology, Kunming 650051, China

## Abstract

In high-dimensional spaces, accuracy and similarity search by low computing and storage costs are always difficult research topics, and there is a balance between efficiency and accuracy. In this paper, we propose a new structure Similar-PBF-PHT to represent items of a set with high dimensions and retrieve accurate and similar items. The Similar-PBF-PHT contains three parts: parallel bloom filters (PBFs), parallel hash tables (PHTs), and a bitmatrix. Experiments show that the Similar-PBF-PHT is effective in membership query and* K*-nearest neighbors (*K*-NN) search. With accurate querying, the Similar-PBF-PHT owns low hit false positive probability (FPP) and acceptable memory costs. With* K*-NN querying, the average overall ratio and rank-*i* ratio of the Hamming distance are accurate and ratios of the Euclidean distance are acceptable. It takes CPU time not I/O times to retrieve accurate and similar items and can deal with different data formats not only numerical values.

## 1. Introduction

In high-dimensional spaces, exact search methods, such as* k*d-tree approaches and* Q*-gram, are only suitable for small size vectors due to huge computation resources. However, similar search algorithms can drastically improve the search speed while maintaining good precision [1], which include VA-files, best-bin-first, space filling curves,* K*-means (see [2] and references therein), NV tree [3],* K*-nearest neighbors (*K*-NN), and locality-sensitive hashing (LSH) [4]. Most* K*-NN methods adopt the Euclidean distance; they assume all coordinates are numerical and own same units and semantics. But, in some applications, the dimension may be string or category, which makes the Euclidean distance questionable and artificial.

In query tools, a bloom filter [5] (BF), as a space-efficient and constant query delay random data structure, has been applied to present a big set and retrieve memberships broadly [6]. But the BF only can present 1-dimensional elements of a set; references [7–9] extended it to present high-dimensional sets and dynamic sets. But these methods can only answer the membership query, not the similarity query. In [10, 11], the LSH functions replace the random hash functions of the BF to implement the similarity search, while [10, 11] only can deal with numerical coordinates and return the elements whose distances from the query are at most CR distance in Euclidean spaces, which lead to false negative probability (FNP).

Here, by computing the Hamming distance, we propose a new structure, called Similar-PBF-PHT, based on the BFs and hash tables (HT) to search the membership as well as the* K*-NN regardless of the radius CR. The Similar-PBF-PHT includes PBFs, PHTs, and a bitmatrix. The PBFs and PHTs apply *d* BFs and HTs to store *d* dimensions, and the bitmatrix stores the dependences of the dimensions. The experiments show that the Similar-PBF-PHT owns better performance in Hamming spaces than other methods. Meanwhile, with* K*-NN searching, it gets a balance performance and can process different data formats while other LSH-based methods can only deal with numerical value.

## 2. Related Work

There are different kinds of approximate search algorithms, and we divide them into three categories to discuss.

The famous one is space partition method, including IDistance [12] and MedRank [13]. The IDistance [12] clusters all high-dimensional elements into multiply spaces and converts them into 1-dimension space. It costs linear space and supports data insertion and deletion; however, if the data distribute uniformly or dimensions are anisotropic, space partition and center selection will be difficult. The MedRank [13] is a rank aggregation and instance optional algorithm, which aggregates the given dataset into *M* sorted lists, where every element has an entry with a form (Id, key). The number of the lists *M* equals log⁡*n*, where *n* is the number of the elements, and by *M* lists probing, the MedRank finds out approximate NN items. The MedRank possesses the best linear-space, but an element insertion or deletion needs update *M* lists and every list requires sorting again.

The LSH and its variants are other famous* K*-NN search algorithms [14], like Rigorous-LSH [15], E2LSH [4, 16], Adhoc-LSH [17], LSB-tree [18], LSB-forest [18], BLSH [11], and so on [19–21]. Let *S* be a set of points in* d*-dimensional space, The Rigorous-LSH [15] applies C-approximate ball cover, which has radius *R* and centers at the query point *q*, denoted as *B*(*q*, *R*). If *B*(*q*, CR) contains at least one point in *S* (*C* ≥ 1 is a constant), it returns a point that is at most CR distance to *q*; others return nothing. The Rigorous-LSH is theoretically perfect, but the query and space costs are expensive. In Euclidean spaces, E2LSH [4, 16] achieves the Rigorous-LSH through* p*-stable distribution [22], which reduces CPU and memory costs of the Rigorous-LSH greatly. The Adhoc-LSH [17] modifies the drawbacks of the Rigorous-LSH by a heuristic approach. Let a query be *q* and a magic radius RM; the Adhoc-LSH returns the points within the radius of RM. If the RM equals the distance between the *q* and the exact NN, the Adhoc-LSH works well. If not, an improper RM may lead to FNP. Beyond locality sensitive hashing (BLSH) [11] scheme uses a two-level hashing algorithm to overcome the lower bound of the FNP [19] and finds CR-NN in Euclidean spaces. Different from other LSH methods, the second-level BLSH, parameterized by different center points, is a data-aware scheme. The outer hash table partitions the data sets into buckets of bounded diameter. For each bucket, the BLSH constructs an inner hash table, which applies the minimum enclosing ball of the points in the bucket as a center point. However, the BLSH still has high memory costs and will bring FNP. The LSB-tree and LSB-forest [18] implement the* K*-NN search by space mapping and* Z*-order coding. By the LSH functions, the LSB-tree [18] first maps *d* dimensional points *S*(*o*) to a lower dimensional points *G*(*o*). Then the LSB-tree gets the* Z*-order [23] value of the *G*(*o*), which is indexed by a conventional B-tree. Multiply LSB-trees form a LSB-forest, which can update efficiently and satisfy query accuracy but space costs are expensive.

The BFs are introduced into the high-dimensional search, like high-dimensional dynamic BFs (MDDBFs) [7], PBF-BF [8], PBF-HT [8], similarity sets [24], and distance-sensitive BFs (DSBF) [10], and so on [25]. The MDDBFs [7] apply *d* parallel standard BFs (PBFs) to present a* d*-dimensional dynamic dataset. By searching *d* PBFs, the MDDBFs find out the membership, but the MDDBFs lack a way to verify the dependency of multiple dimensions of an item, which causes high FPPs with membership retrieval. To reduce the FPP, PBF-BF and PBF-HT [8] add another BF and hash table (HT) to the PBFs to store the verification value of the different dimensions. However the methods above based on the BFs can only answer the membership query, not similarity query.

Distance-sensitive BFs (DSBF) [10] replace the uniform hash functions in the BF with the LSH functions to find out similar strings. But the DSBF can only differentiate a query string that differs from all strings in the dataset on a (constant) *δ*-fraction of bits. The locality-sensitive bloom filter (LSBF) [26] uses two-level BFs to implement the approximate item query. The first-level bloom replaces the random hash functions with locality-sensitive hash function (LSH), which is based on* p*-stable distribution [22], and maps all items to *m* bit-bloom arrays. To keep the integrity and reduce the FPP, the second level BF stores the hash verification signature formed by the *k* LSH functions in the first-level BF. In order to reduce the FNP, the LSBF needs to probe the neighbor bits in the first-level BF, which leads to cost more query time. Meanwhile since the LSH function concentrates most points around the mean and maps some neighboring points to remote bits, it will bring bigger FPP and FNP.

## 3. Structures and Working Mechanism

### 3.1. Structures

**Figure 1:**
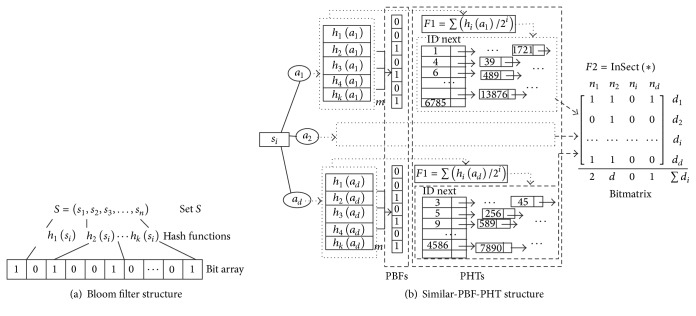
Bloom filter and Similar-PBF-PHT.

A standard BF [5] applies an array of *m* bits (initially all are set to 0) and *k* independent hash functions *h*_*i*_ to represent a set *s* = {*a*_1_, *a*_2_,…, *a*_*n*_} of *n* elements, as shown in Figure 1(a). If an element is mapped into the BF by *h*_*i*_, the corresponding bit *h*_*i*_(*a*_*i*_)%*m* is set to 1. Given a query *q*, by *k* hash functions *h*_*i*_(*q*)%*m* mapping, the BF answers whether the *q* is a member of *S* with a FPP. In order to support elements deletion, counting bloom filter (CBF) [27, 28] replaces the array of *m* bits with *m* counters.

In this paper, to present high-dimensional elements, *d* parallel BFs (PBFs) and parallel hash tables (PHTs) are proposed to represent the elements with *d* dimensions. At the same time, a bitmatrix is introduced to keep the inherent dependency of the dimensions and reduce the FPP, as shown in Figure 1(b).

#### 3.1.1. PBFs

To store the dimensions, this paper introduced *d* BFs (Figure 1(b)), and every BF owns *k* independent hash functions [29] *h*_1_, *h*_2_,…, *h*_*k*_ and a bit array with *m* length, denoted as *B*. Let *a*_*j*_ present the* j*th dimension and *h*[*j*][*i*](*a*_*j*_) present* i*th hash function value of the* j*th dimension. When *a*_*j*_ is mapped into *B*_*j*_, the corresponding place *B*_*j*_[*h*[*j*][*i*](*a*_*j*_)%*m*] is set to 1. Since attenuation method can make hash values distribute broadly [8] and reduce the FPP, we apply the attenuation sum of *k* hash values *F*(1) = ∑*h*[*j*][*i*](*a*_*j*_)/2^*i*^ to store the verification value of the dimension *a*_*j*_.

#### 3.1.2. PHTs

In order to find out which dimensions and how many dimensions of the elements in set *S* are similar to the query *q*, this paper utilizes *d* parallel hash tables (PHTs) and hash links to store identifications (IDs) of the elements. Each hash table, denoted as HT, is indeed a link array with *m*_2_ length.

#### 3.1.3. Bitmatrix

Since *d* dimensions are stored into *d* BFs and HTs separately, the integrity of the elements is destroyed, which leads to query confusion. Thus, an auxiliary structure, called bitmatrix, is added to record dimensions hit in the PBFs and PHTs. After *d* dimensions are checked in the PBFs and PHTs, numbers of the hit dimensions are summed up in the bitmatrix; that is, *F*(2) = max⁡(∑_*i*=1_^*d*^ *d*_*i*_). If *F*(2) = *d*, the query is a member of the set *S* with a FPP, as shown in (1). If *F*(2) = 0, no dimension of the query is in the set, for example, (2). If 0 < *F*(2) < *d* the query is a similar elements with a FPP, as shown in (3).

*The Bitmatrix*

- Membership:
  (1) $$\begin{array}{c} \begin{array}{c} \;\\ d_{1}\\ d_{2}\\ d_{3}\\ d_{4}\\ d_{5}\\ \sum \;d_{i} \end{array}\begin{array}{cccccccc} n_{1} & n_{2} & n_{3} & n_{4} & n_{5} & n_{6} & n_{7} & n_{8}\\ \left[\begin{array}{c} 0\\ 1\\ 1\\ 0\\ 1 \end{array}\right. & \begin{array}{c} 1\\ 0\\ 0\\ 1\\ 1 \end{array} & \begin{array}{c} 0\\ 0\\ 0\\ 1\\ 0 \end{array} & \begin{array}{c} 1\\ 0\\ 0\\ 1\\ 0 \end{array} & \begin{array}{c} 1\\ 1\\ 1\\ 1\\ 1 \end{array} & \begin{array}{c} 0\\ 0\\ 0\\ 0\\ 1 \end{array} & \begin{array}{c} 0\\ 0\\ 0\\ 0\\ 0 \end{array} & \left.\begin{array}{c} 0\\ 0\\ 0\\ 0\\ 0 \end{array}\right]\\ 3 & 3 & 1 & 2 & 5 & 1 & 0 & 0 \end{array} \end{array}$$
- Outlier:
  (2) $$\begin{array}{c} \begin{array}{c} \;\\ d_{1}\\ d_{2}\\ d_{3}\\ d_{4}\\ d_{5}\\ \sum \;d_{i} \end{array}\begin{array}{cccccccc} n_{1} & n_{2} & n_{3} & n_{4} & n_{5} & n_{6} & n_{7} & n_{8}\\ \left[\begin{array}{c} 0\\ 0\\ 0\\ 0\\ 0 \end{array}\right. & \begin{array}{c} 0\\ 0\\ 0\\ 0\\ 0 \end{array} & \begin{array}{c} 0\\ 0\\ 0\\ 0\\ 0 \end{array} & \begin{array}{c} 0\\ 0\\ 0\\ 0\\ 0 \end{array} & \begin{array}{c} 0\\ 0\\ 0\\ 0\\ 0 \end{array} & \begin{array}{c} 0\\ 0\\ 0\\ 0\\ 0 \end{array} & \begin{array}{c} 0\\ 0\\ 0\\ 0\\ 0 \end{array} & \left.\begin{array}{c} 0\\ 0\\ 0\\ 0\\ 0 \end{array}\right]\\ 0 & 0 & 0 & 0 & 0 & 0 & 0 & 0 \end{array} \end{array}$$
- Similarity:
  (3) $$\begin{array}{c} \begin{array}{c} \;\\ d_{1}\\ d_{2}\\ d_{3}\\ d_{4}\\ d_{5}\\ \sum \;d_{i} \end{array}\begin{array}{cccccccc} n_{1} & n_{2} & n_{3} & n_{4} & n_{5} & n_{6} & n_{7} & n_{8}\\ \left[\begin{array}{c} 0\\ 1\\ 1\\ 0\\ 1 \end{array}\right. & \begin{array}{c} 1\\ 0\\ 0\\ 1\\ 1 \end{array} & \begin{array}{c} 0\\ 0\\ 0\\ 1\\ 0 \end{array} & \begin{array}{c} 1\\ 0\\ 0\\ 1\\ 0 \end{array} & \begin{array}{c} 1\\ 0\\ 1\\ 1\\ 1 \end{array} & \begin{array}{c} 0\\ 0\\ 0\\ 0\\ 1 \end{array} & \begin{array}{c} 0\\ 0\\ 0\\ 0\\ 0 \end{array} & \left.\begin{array}{c} 0\\ 0\\ 0\\ 0\\ 0 \end{array}\right]\\ 3 & 3 & 1 & 2 & 4 & 1 & 0 & 0 \end{array} \end{array}$$

### 3.2. Working Mechanism

#### 3.2.1. Initialization or Insertion

When *d* dimensions of an element are mapped into *d* BFs and HTs by *k* hash functions, the locations of the bit array in the PBFs are set to 1, and the attenuation hash values are summed up: *F*(1) = ∑_*i*=1_^*k*^ *h*[*j*][*i*](*a*_*j*_)/2^*i*^. By *F*(1)%*m*_2_ mapping, the corresponding link in the* j*th HT is found, and a new hash node is added to the tail of the link to store the item's ID.

#### 3.2.2. Query

Only when the dimension returns 1 in the BF will the attenuated hash values be summed up and located in the corresponding HT. The hit elements' IDs are found and the corresponding bits in the bitmatrix are set to 1. After all *d* dimensions are mapped, columns in the bitmatrix are summed up. If the summation is in the range between 1 and *d*, the membership or similar elements are obtained.

#### 3.2.3. Element Deletion

Since bit deletion in a BF will bring FPP; the Similar-PBF-PHT only needs to delete the hash node in the corresponding HT.

## 4. Performance Analysis

Since the BF only has FPP but not FNP [24], we evaluate the performance of the Similar-PBF-PHT by the quality of results, FPP, query time, and space consumption.

### 4.1. False Positive Probability (FPP)

*False Positive.* A query *q* is a false positive to object set *S*, if the query gets a positive answer while in fact *q* is not a membership, or there does not exit a neighbor object *p* in Euclidean or Hamming spaces. FPP is the probability of false positives.

#### 4.1.1. FPP of a BF and a HT

**Theorem 1 .**

Where *n* elements in the set *S* have been mapped to a BF with *m* bits by *k* different independent hash functions, the FPP of a BF [24] is

(4) FPPBF1−p′k=1−1−1mknk≈1−e−kn/mk.

When *k* = (*m*/*n*)ln⁡2, the *f*_*BF*_ obtains the minimum value (1/2)^*k*^ or 0.6185^*m*/*n*^ [24].

**Theorem 2 .**

A HT possess $f_{{HT}_{max}}=2\phi \left(\left(\sqrt{3}*2^{k}n\right)/2\left(q-1\right)\sqrt{k}\right)-1$ upper bound FPP, if *k* hashes of the BF follow uniform distribution and the attenuated check value *v*(*a*_*j*_) = ∑_*i*=1_^*k*^ *h*_*i*_(*a*_*j*_)/2^*i*^ obeys normal distribution.

**Proof**

In this paper, *d* BFs are used to present the *d* dimensions of elements, and each BF owns *k* independent hash functions [19]. Let each random variable *h*_*i*_(*a*_*j*_) follow the uniform distribution with range {1,…, *M*}, the expected value of (1 + *M*)/2, and variance of (*M* − 1)^2^/12, and *v*(*a*_*j*_) = ∑_*i*=1_^*k*^ *h*_*i*_(*a*_*j*_) is ranged {*k*,…, *kM*}. According to central limit theorem [1], if *k* is big enough, the random variable *v*(*a*_*j*_) satisfies a normal distribution with the expected value of *k*(1 + *M*)/2 and variance of *k*(*M* − 1)^2^/12. Because the sum of attenuated value of *k* hash functions *v*(*a*_*j*_) = ∑_*i*=1_^*k*^ *h*_*i*_(*a*_*j*_)/2^*i*^ can reduce the FPP, we store the attenuated value of* j*th dimension of element *a* into the* j*th HT. It is difficult to estimate the probability density functions of *v*(*a*_*j*_), and according to birthday attack [2], when *v*(*a*_*j*_) distributes uniformly, the collision will be minimum. For simplicity, we suppose *v*(*a*_*j*_) satisfies normal distribution to estimate the upper bound of false positive.

Let *v*′(*a*_*j*_) = ∑_*i*=1_^*k*^ *h*_*i*_(*a*_*j*_)/2^*k*^; due to ∑_*i*=1_^*k*^ *h*_*i*_(*a*_*j*_)/2^*i*^ > ∑_*i*=1_^*k*^ *h*_*i*_(*a*_*j*_)/2^*k*^, the discrete degree of *v*(*a*_*j*_) is bigger than *v*′(*a*_*j*_), while the collision probability of *v*(*a*_*j*_) is smaller than *v*′(*a*_*j*_). Let *p*(*v*(*x*_*j*_)) be the FPP of the verification value of the* j*th attribute of* x*. There exists *p*(*v*(*x*_*j*_)) < *p*(*v*′(*x*_*j*_)). According to central limit theorem [30], *v*′(*a*_*j*_) = ∑_*i*=1_^*k*^ *h*_*i*_(*a*_*j*_)/2^*k*^ satisfy normal distribution, with the expected value of *μ* = *k*(1 + *M*)/(2*∗*2^*k*^) and variance of *δ* ^2^ = *k*(*M* − 1)^2^/(12*∗*2^*k*^).

Let there be *n* items and *v*′(*a*_*j*_) be in the range of {1,…, *M*_1_}. In order to get the upper bound, we compute the maximum FPP of each dimension, that is, *f*_HT_max__:

(5) fHTmax⁡pvaj≤pv′aj=pμ−n2≤v′aj≤μ+n2=2ϕn2δ−1=2ϕ3∗2kn2q−1k−1.

#### 4.1.2. FPP of Similar-PBF-PHT

**Theorem 3 .**

With* K*-NN searching, the average FPP of *i* dimensions misdetected, *f*_*similar*-*PBF*-*PHT*_^*i*^ is ((1 + *f*_*BF*-*HT*_)^*i*^ − 1)/(2^*i*^ − 1), in which *f*_*BF*-*HT*_ is the FPP of a dimension misdetected in both the BF and HT. When *i* = *d*, it is membership search, and the hit FPP *f*_*Similar*-*PBF*-*PHT*_^*hit*^ is ((1 + *f*_*BF*-*HT*_)^*d*^ − 1)/(2^*d*^ − 1).

**Proof**

The BF and HT are independent, so the FPP of a dimension is *f*_BF-HT_ = *f*_BF_ *∗f*_HT_. With similarity querying, if any *i* dimensions collide in the BFs and HTs and other *i* − 1 dimensions are members, the collision happens, which satisfies binomial distribution, and all combinations are

(6) Tccdi−1cd−i+11+cdi−2cd−i+22+⋯+cd0cdi=d!d−i!∑j=0i1k−j!j!−cdicd−i0=cdi2i−1.

With similarity searching, let *f* = *f*_BF-HT_, and the average FPP *f*_Similar-PBF-PHT_^*i*^ is

(7) $$\begin{array}{c} f_{\text{similar-PBF-PHT}}^{i}=\frac{1}{T_{c}}\left(\;c_{d}^{i-1}c_{d-i+1}^{1}f^{1}+\cdots +c_{d}^{0}c_{d}^{i}f^{i}\;\right). \end{array}$$

When 0 ≤ *f* ≤ 1, there is *C*_*i*_^0^ *f* ^0^ + *C*_*i*_^1^ *f* ^1^ + ⋯+*C*_*i*_^*i*^ *f* ^*i*^ = (1 + *f*)^*i*^, and the FPP is

(8) fsimilar-PBF-PHTi1Tccdi−1cd−i+11f1+⋯+cd0cdifi=1Tccdici1f1+ci2f2+⋯+ciifi=1+fi−12i−1.

When *i* = *d*, it is the membership search, namely, *f* ^hit^.

If *d* dimensions are misdetected simultaneously, *f*_BF-HB_^*d*^ gets the minimum value. If only one dimension is falsely detected, *f* ^hit^ is the maximum value*f*_BF-HB_. There is

(9) $$\begin{array}{c} f_{\text{BF-HT}}^{d}\leq f_{\text{similar-PBF-PHT}}^{\text{hit}}\leq f_{\text{BF-HT}}. \end{array}$$

**Figure 2:**
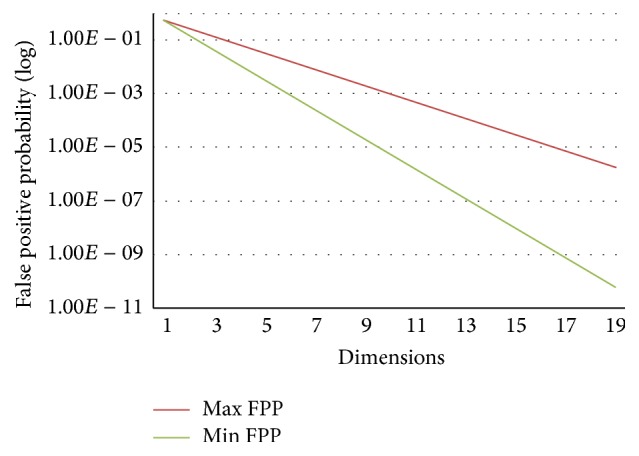
The maximum and minimum value of log⁡*f* ^hit^.

In Figure 2, let *f*_BF-HT_ = 0.5; with dimensions increasing, the maximum and minimum of the *f*_similar-PBF-PHT_^hit^ decay exponentially.

### 4.2. Average Overall Ratio

We evaluate the quality of a* K*-NN search result by rank-*i* ratio and average overall ratio (AOR) [18], which are used in most experiments. The rank-*i* ratio is denoted by *R*_*i*_(*q*) and defined as

(10) $$\begin{array}{c} R_{i}\left(\;q\;\right)=\frac{\left\|o_{i},q\right\|}{\left\|{o_{i}}^{*},q\right\|}, \end{array}$$

where *i* ∈ [1, *K*], ‖*o*_*i*_, *q*‖ is the distance of the queried* i*th neighbor to *q*, and ‖*o*_*i*_^*∗*^, *q*‖ is the distance of the actual* i*th neighbor to *q*. The overall approximation ratio is the mean of the ratios of all ranks, namely, (∑_*i*=1_^*K*^ *R*_*i*_(*q*))/*K*.

### 4.3. Complexity

#### 4.3.1. Storage Space

The storage spaces of the Similar-PBF-PHT contain three parts:

(i) When the FPP of a BF is not greater than *ε* and the number of hash functions is  optimal, to express the set *S* of *n* elements, the size of the BF array must be  *m* ≥ *n* (log_2_⁡(1/*ε*)/ln⁡2) = −*n* log_2_⁡*e* × log_2_⁡*ε*. Then the spaces required by *d* parallel BFs are   (bits)

(11) $$\begin{array}{c} m\geq dn\frac{\log_{2}\left(1/\varepsilon \right)}{\ln2}=\left(\;-\log_{2}e\;\log_{2}\varepsilon \;\right)dn. \end{array}$$

(ii) A HT needs to store all IDs of the elements and the next node. Let *αn* be the  length of the HT (0 < *α*), a node takes up *l*_1_ bits, and the HT requires spaces with a range  from *nl*_1_ to (*α∗n* + *n*)*l*_1_. The spaces range of the *d* HTs is [*ndl*_1_, *nd*(1 + *α*)*l*_1_] bits.

(iii) The bitmatrix needs *nd* bits.

The storage spaces of the Similar-PBF-PHT are (bytes)

(12) Space−nd log2⁡e log2⁡ε+nd1+αl1+nd8=nd8−log2⁡e log2⁡ε+1+αl1+1.

 When *α* = 0, space gets the minimum value. and when *l*_1_, *ε*, and *α* are constants, the space complexity of the Similar-PBF-PHT is

(13) $$\begin{array}{c} O\left(\;c_{1}nd\;\right). \end{array}$$

#### 4.3.2. Search Time

When querying, the PBFs need *kd* times hash calculation, the PHTs require *dl*_2_ times to search the IDs, in which *l*_2_ is the average length of the HT bucket links.

During the membership searching, all hit element's IDs need to be recorded, and the time complexity is

(14) $$\begin{array}{c} O\left(\;dk+dl_{2}\;\right). \end{array}$$

The bitmatrix traverses at most *K* times. So the time complexity is

(15) $$\begin{array}{c} O\left(\;dk+dl_{2}+K\;\right). \end{array}$$

## 5. Experiments

### 5.1. Dataset and Setting

The BF is designed to represent a set, and there is no benchmark. Here we choose 4 datasets used in most experiments; they are Color [13], Mnist [12], Varden [18], and Reuters 21578 [31]. Data formats in the Reuters 21578 are various including digital, character, symbols, and their combinations. We use it to generate 49396-item dataset with 1000 dimensions to test the performance of the Similar-PBF-PHT, including the query latency and the ability of data processing. The experiments run on a computer with 2.5 GHz Intel double Core processors and 8 G RAM.

### 5.2. Membership Query

In this section, we will discuss performances of different methods in membership query.

**Figure 3:**
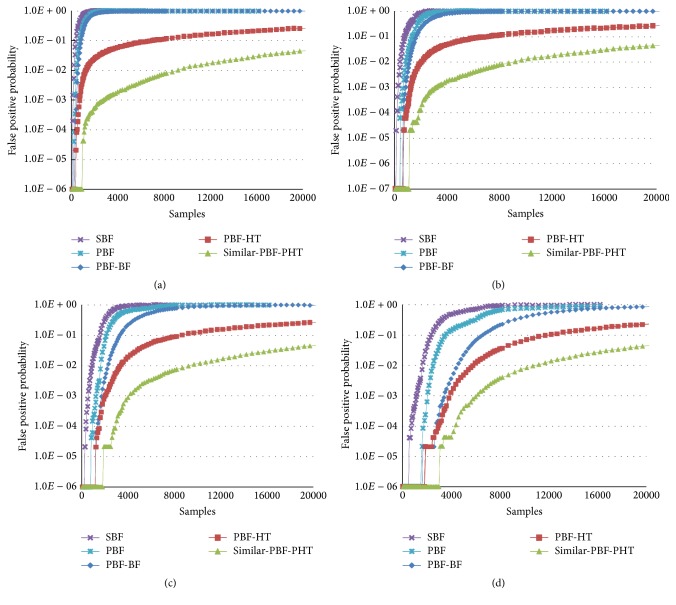
FPPs of the SBF, PBF, PBF-HT, PBF-BF, and the Similar-PBF-PHT under 3 dimensions. (a) Address space *m* = 320. (b) Address space *m* = 640. (c) Address space *m* = 1280. (d) Address space *m* = 2560.

Let *d* = 3, *m* = 320(640, 1280, 2560), = 5, *α* = 1.1, and *l*_*v*_ = 32, where *l*_*v*_ is bits of every verification values in the PBF-HT and Similar-PBF-PHT.  Figure 3 displays the FPPs of the SBF [5], PBF [7], PBF-HT [8], PBF-BF [8], and Similar-PBF-PHT on Reuters 21578 data. With the *m* increasing, the FPPs decrease, and to a constant *m*, the FPPs will increase with the number of the elements growing, especially the SBF and PBF. When the number of the items exceeds a threshold (*n* ≥ *m*), the FPPs of the SBF and PBF are nearly equal to 1, which is consistent in the BF theory. In different *m*, the Similar-PBF-PHT gets the lowest FPP; even when *m* = 320, the biggest FPP is not beyond 0.01, while the FPPs of others are almost 1.

**Figure 4:**
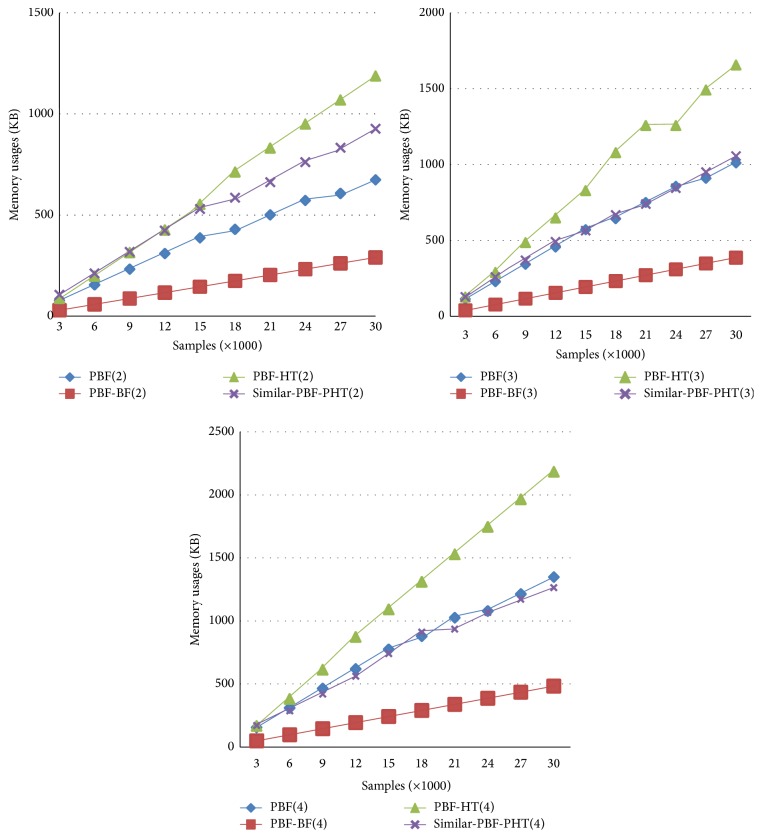
Memory usages of the PBF, PBF-BF, PBF-HT, and the Similar-PBF-PHT.

Figure 4 demonstrates memory usages of the PBF, PBF-BF, PBF-HT, and Similar-PBF-PHT on the Reuters 21578 dataset, when FPP = 0.00098, *d* = 4, *k* = 5, *n* = [3000, 30000], and *l*_*v*_ = 32. According to formula (13), to fit a constant FPP, the memory usage will grow with the number of the items increasing. The hash tables and the bitmatrix reduce the FPP at the cost of memory, and the BF's bits arrays take up 1/4 spaces of the CBFs in other 3 schemes. All these make the space consumption of the Similar-PBF-PHT just a little higher than the PBF but lower than the PBF-HT.

### 5.3. *K*-NN Search

**Figure 5:**
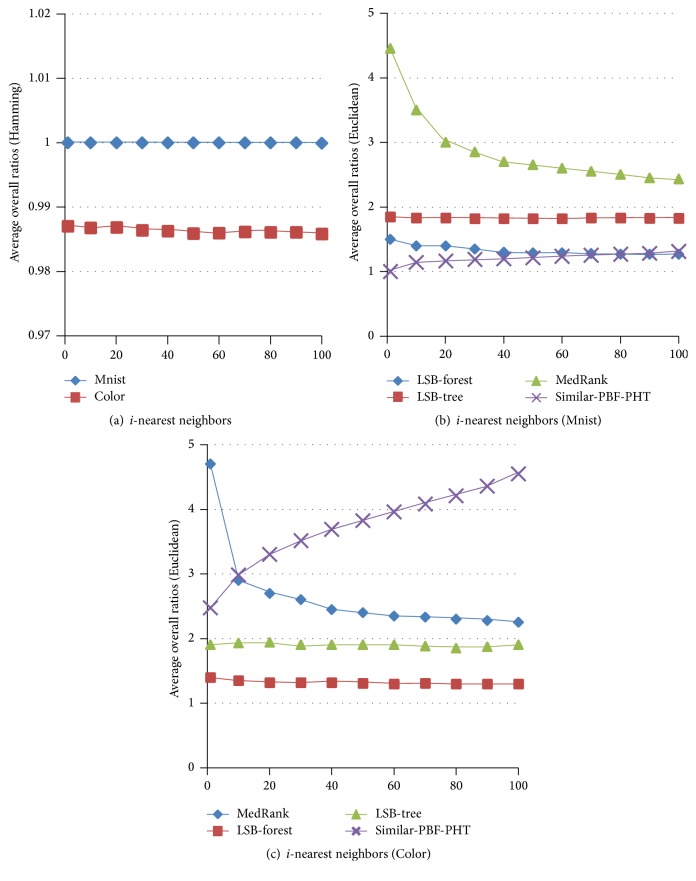
Average overall ratios of [1–100]-NN on Color and Mnist.

To evaluate the accuracy of the* K*-NN search, we compare the average overall ratios of the Rigorous-LSH [15], MedRank [13], Adhoc-LSH [17], LSB-tree, and LSB-forest [18] with the Similar-PBF-PHT on the Color and Mnist dataset, as shown in Figure 5. Workload is set to 50, and 1–100 nearest neighbors are searched. In Hamming spaces, the ratios of the Similar-PBF-PHT are almost equal to 1. In Euclidean spaces, the ratios of the Similar-PBF-PHT are not stable; the overall ratios on the Mnist are almost as good as the LSB-forest, but the ratios on Color are a little higher and increase with the number of nearest neighbors. The main reasons are that the dimensions of the Mnist are sparse (most values are 0), and most Hamming distances are 0. While the dimensions of the Color are dense, a small distance (0.0001) in Euclidean spaces will be recognized as 1 in Hamming spaces. All these make the accuracy decrease, but the ratios are still beyond 0.98.

**Figure 6:**
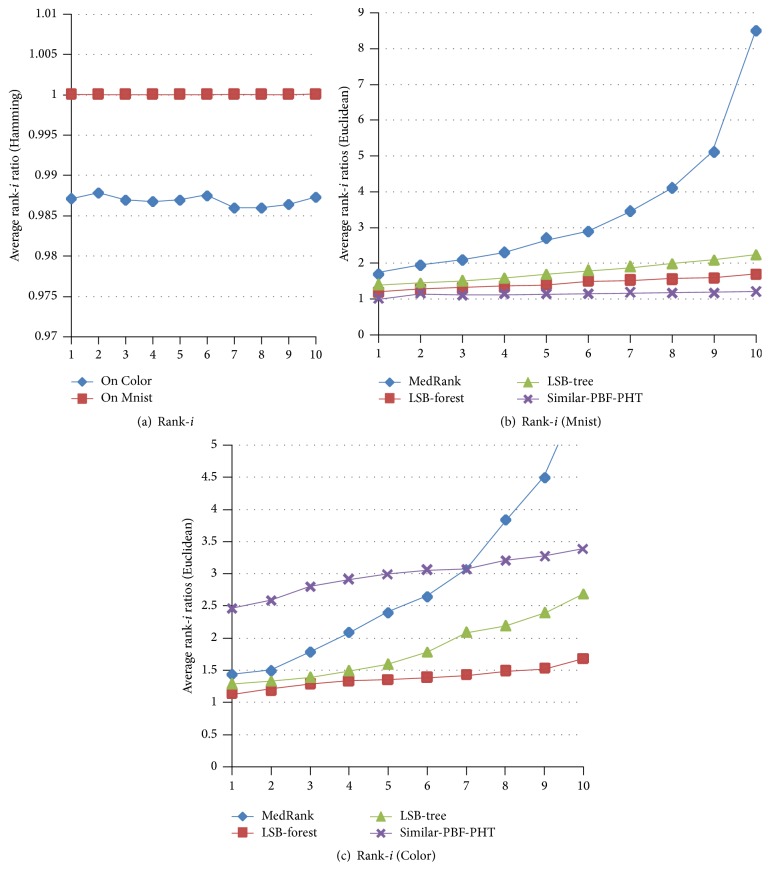
Average rank-*i* ratios of [1–10]-NN on Color and Mnist.

Figure 6 displays average rank-*i* ratios of the Euclidean and Hamming distance. ‖*o*_*i*_, *q*‖ presents Hamming distance; because of the FPP of the BF, the actual distance is less than the query distance; there exists *R*_*i*_(*q*) ≤ 1. In Figure 6(a), with *i* increasing, the rank-*i* ratios of Hamming distance are stable and not lower than 0.985. On the Mnist (Figure 6(b)), rank-*i* ratios of Euclidean distances of the Similar-PBF-PHT are minimum, almost equal to 1, while, on the Color (Figure 6(c)), the ratios of Euclidean distance increase slowly and are higher than the LSB-tree and LSB-forest's; when the rank − *i* ≥ 7, it becomes lower than the MedRank.

Table 1 analyzes the memory consumption (MB) on the Varden dataset, setting *B* = 4096 bytes, *c* = 2, and $l=\sqrt{dn/B}$. Although the memory costs of the LSB-tree are less, its FPPs are higher than the LSB-forest, so we abandon it. The memory usages of the Similar-PBF-PHT are minimum while the Rigorous-LSH are maximum, and all consumption increases with *d* and *n*. When *c* = 2 the memory costs of the LSB-Forest are almost as big as the Adhoc-LSH.

**Figure 7:**
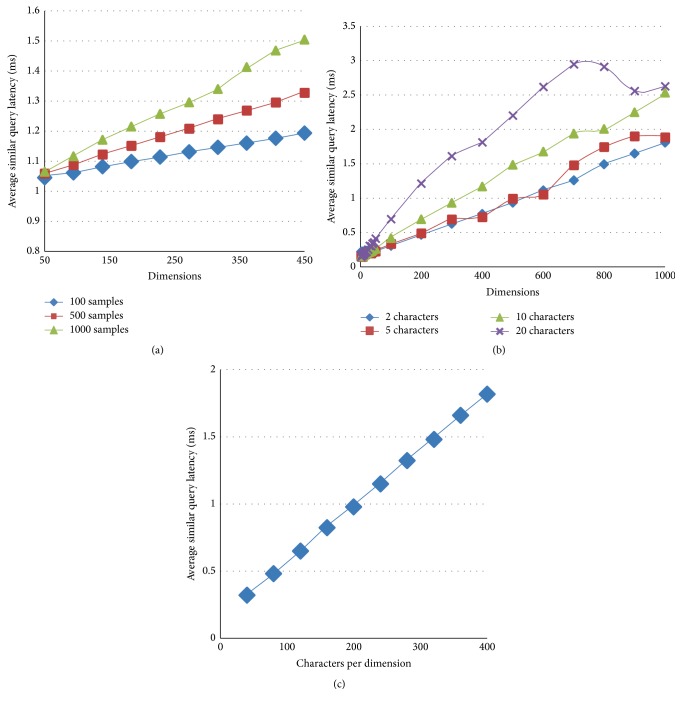
Average query latency of Similar-PBF-PHT when searching 10-NN. (a) Average query latency of Similar-PBF-PHT when samples* n* are 100, 500, and 1000. (b) Average similar query latency when *d* ∈ [2–1000], *n* = 5000, and *k* = 5. (c) Average similar query latency when the length of the dimension is within the range [40–400], *d* = 50, *k* = 5, and *n* = 5000.

The Similar-PBF-PHT can deal the dimensions with different formats and lengths, and the length of dimension and number of samples will affect the query time. In Figure 7(a), we set *n* to 100, 500, and 1000, respectively, and every dimension contains 20 characters (big enough to most applications) to search 10-NN. With dimensions growing, the average query latency of the Similar-PBF-PHT increases linearly. Let *n* = 5000, *K* = 10, and *K* = 10; Figures 7(b) and 7(c) demonstrate effects of different dimension's lengths on query delay with 10-NN searching. Average query latencies will increase with the numbers of the characters and dimensions. This is because most of the CPU time is wasted on processing the hash values of the characters.

**Figure 8:**
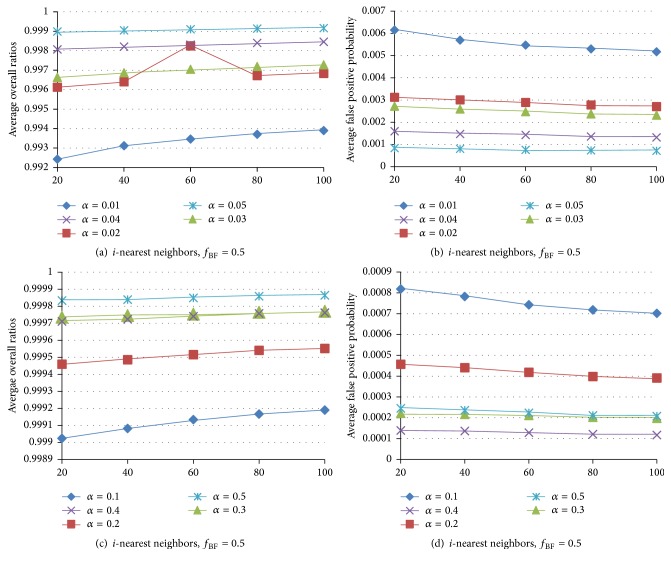
Average overall ratios and average FPP when searching [20–100]-NN on Color under different *f*_BF_ and *α*.

In Figure 8, we analyze the effects of the parameters *α* and *f*_BF_ on the AORs and FPPs of the Similar-PBF-PHT. Let *n* = 50000, *k* = 3, *l*_*v*_ = 32, and  *K* ∈ [20–100]-NN and let test workload be 10000. As shown in the Figure 8, under different *α*, even small *α* (0.01), and big *f*_BF_ (0.5), the Similar-PBF-PHT gets good query results and low FPPs. That means the PHT and the bitmatrix can effectively improve the detection accuracy. *α* affects the query accuracy much more than the FPP of the BF. With *α* increasing, the FPP decreases and the AOR increases; at the same time the space consumption increases.

## 6. Conclusions

In this paper, we propose a comprehensive structure, called Similar-PBF-PHT, to represent and search member and similar elements of a big dataset in high-dimensional spaces by computing Hamming distance. We analyze its working mechanism, FPP, and space and time complexity in detail. The experiments show that, with membership searching, compared with the PBF, PBF-HT, and PBF-BF, the Similar-PBF-PHT owns lower hit FPP by a low memory cost. The Similar-PBF-PHT costs less storage than the schemes based on the locality sensitive hash, including the Rigorous-LSH, LSB-forest, Adhoc-LSH, and BLSH. With* K*-NN items querying, it costs CPU time, not I/O times, which make it have less query latency. Meanwhile, the Similar-PBF-PHT computes hash values of all characters in each dimension, so it can deal with different data formats (chars, number, symbol, and so on), and the number of characters will affect the query time. The average overall ratios (query accuracy) and the average rank-*i* ratios of the Hamming distance are accurate. All these advantages make it appropriate for representing and searching items in high-dimensional spaces, such as database and documents similar search.

Although the Similar-PBF-PHT can get good performance in Hamming spaces, memory costs and the FPP of Euclidean spaces for* K*-NN searching are still a little higher. In the future, we will study the local sensitive hash functions to replace the random hash functions and further reduce the storage spaces.

## Figures and Tables

**Table 1a:** (a) Space versus cardinality *n*  (*d* = 50)

*n*	10000	25000	50000	75000	100000
Rigorous-LSH	895	3624	10323	18892	29016
Adhoc-LSH	57	231	660	1208	1855
LSB-forest	57	231	660	1208	1855
Similar-PBF-PHT	13	31	61	92	121

**Table 1b:** (b) Space versus dimensionality *d* (*n* = 50000)

*d*	25	50	75	100
Rigorous-LSH	382	896	1563	2436
Adhoc-LSH	24	57	101	159
LSB-forest	24	57	101	159
Similar-PBF-PHT	31	61	93	120
